# Association between Diet Quality Indices and Incidence of Type 2 Diabetes in the Melbourne Collaborative Cohort Study

**DOI:** 10.3390/nu13114162

**Published:** 2021-11-20

**Authors:** Allison M. Hodge, Md Nazmul Karim, James R. Hébert, Nitin Shivappa, Barbora de Courten

**Affiliations:** 1 Cancer Epidemiology Division, Cancer Council Victoria, Melbourne, VIC 3004, Australia; 2 Centre for Epidemiology and Biostatistics, Melbourne School of Population and Global Health, University of Melbourne, Parkville, VIC 3010, Australia; 3 School of Public Health and Preventive Medicine, Monash University, Melbourne, VIC 3004, Australia; nazmul.karim@monash.edu; 4 Cancer Prevention and Control Program, University of South Carolina, Columbia, SC 29208, USA; jhebert@mailbox.sc.edu (J.R.H.); shivappa@email.sc.edu (N.S.); 5 Department of Epidemiology and Biostatistics, Arnold School of Public Health, University of South Carolina, Columbia, SC 29208, USA; 6 Department of Medicine, School of Clinical Sciences, Monash University, Melbourne, VIC 3168, Australia; barbora.decourten@monash.edu

**Keywords:** type 2 diabetes, obesity, inflammation, diet, insulin resistance

## Abstract

Type 2 diabetes mellitus is a common condition whose incidence is increasing worldwide, and for which obesity and diet are important risk factors. The aim of this study was to assess the association of three diet quality scores with diabetes risk and how much of the association was mediated through body size. The Melbourne Collaborative Cohort Study recruited 41,513 men and women aged 40–69 years during 1990–1994. At baseline, data were collected on lifestyle and diet, anthropometric measures were performed. Incident diabetes was assessed by self-report at follow-up surveys in 1994–1998 and 2003–2007. The associations between the dietary inflammatory index (DII^®^), Mediterranean Diet Score (MDS) and the Alternative Healthy Eating Index—2010 and incident diabetes were assessed using Poisson regression, adjusting for age, sex, physical activity, smoking, alcohol consumption, socio-economic status (area based) and family history of diabetes. Data from 39,185 participants were included in the analysis and 1989 cases of diabetes were identified. Both DII and AHEI-2010 were associated with diabetes incidence, but MDS was not. In the top quintile of DII (most pro-inflammatory) vs. the least inflammatory quintile IRR was 1.49 95% CI (1.30, 1.72), *p* trend across quintiles <0.001. For AHEI-2010 the IRR was 0.67 (0.58, 0.78), *p* trend <0.001 for the healthiest vs. the least healthy quintile. Mediation analysis indicated that body size (body mass index/waist to hip ratio) mediated 35–48% of the association with incident diabetes for the AHEI and DII. Healthier diets may reduce risk of diabetes both by reducing weight gain and other mechanisms such as reducing inflammation.

## 1. Introduction

Diabetes had an estimated prevalence of 9.3% worldwide (463 million people) and caused 4 million deaths in 2019, thus posing a significant health and economic burden to society [1]. Obesity is a major risk factor for type 2 diabetes mellitus (which typically occurs later in life). It has been estimated that 53% of the burden of diabetes was attributable to overweight and obesity in Australia [2]. Many randomised controlled trials have shown that lifestyle interventions consisting of healthy diet and physical activity in people at increased risk for type 2 diabetes delay onset of diabetes [3], largely due to weight loss [4]. Diet is a crucial factor driving weight loss as it is very difficult to overcome the effect of energy-dense diets with mild to moderate exercise [5].

There are many dietary indices used to evaluate overall diet quality. Variations of Mediterranean Diet Scores (MDS) have been around since 1995, and stronger adherence to these eating patterns has been found to be associated with reduced risk of diabetes [6,7]. Diets following the Healthy Eating Index (HEI) or Alternative Healthy Eating Index (AHEI-2010) and Dietary Approaches to Stop Hypertension (DASH) patterns also have been found to be associated with reduced diabetes risk [6]. An analysis of data from the Singapore Chinese Health Study found that diets classified as the healthiest according to alternative Mediterranean Diet score (aMED), AHEI-2010, DASH, overall-plant-based and healthy plant-based were all associated with lower risk of diabetes incidence, ranging from 16% less for aMED to 29% less for DASH [8], but the analysis did not specifically test whether one was better than the others.

Chronic low-grade inflammation has been associated with higher incidence of diabetes through worsening of insulin resistance [9,10] and it has been shown that diet can effectively influence inflammation as assessed by C-reactive protein in a meta-analysis of RCTs [11]. Analysis of long-term data from the PREDIMED study showed that reductions in inflammatory biomarkers were independent of changes in body mass index (BMI) and waist circumference [12]. Furthermore, it has been found that diabetes risk was higher in men and women scoring in the highest quintile of an Empirical Dietary Inflammatory Pattern derived to best predict circulating concentrations of inflammatory biomarkers (C reactive protein (CRP), interleukin-6 (IL-6) and tumour necrosis factor a receptor 2 (TNF-αR2)), relative to the lowest, least inflammatory quintile [13,14]. Several cross-sectional studies have shown that a high dietary inflammatory index (DII^®^) score (indicating a more pro-inflammatory diet) is associated with insulin resistance, insulin secretory dysfunction and presence of diabetes [15]. In addition, high DII was associated with the development of diabetes in women prospectively. Only one case-control study of prediabetes included men, showing a more pro-inflammatory diet was associated with higher risk of the outcome [15].

We have previously examined three dietary indices (DII, MDS and AHEI-2010) and development of obesity and showed that healthier diets assessed by the three scores were associated with lower waist to hip ratio (WHR), but MDS was not associated with future BMI [16]. AHEI-2010 was assessed as being the most strongly associated with future weight. In this study, we aim to examine whether these diet scores are associated with diabetes risk, whether this is mediated by body size, and whether there is any evidence that the association is stronger for one than the other dietary scores using observational data from the Melbourne Collaborative Cohort Study (MCCS).

## 2. Materials and Methods

### 2.1. The Melbourne Collaborative Cohort Study (MCCS)

**Figure 1:**
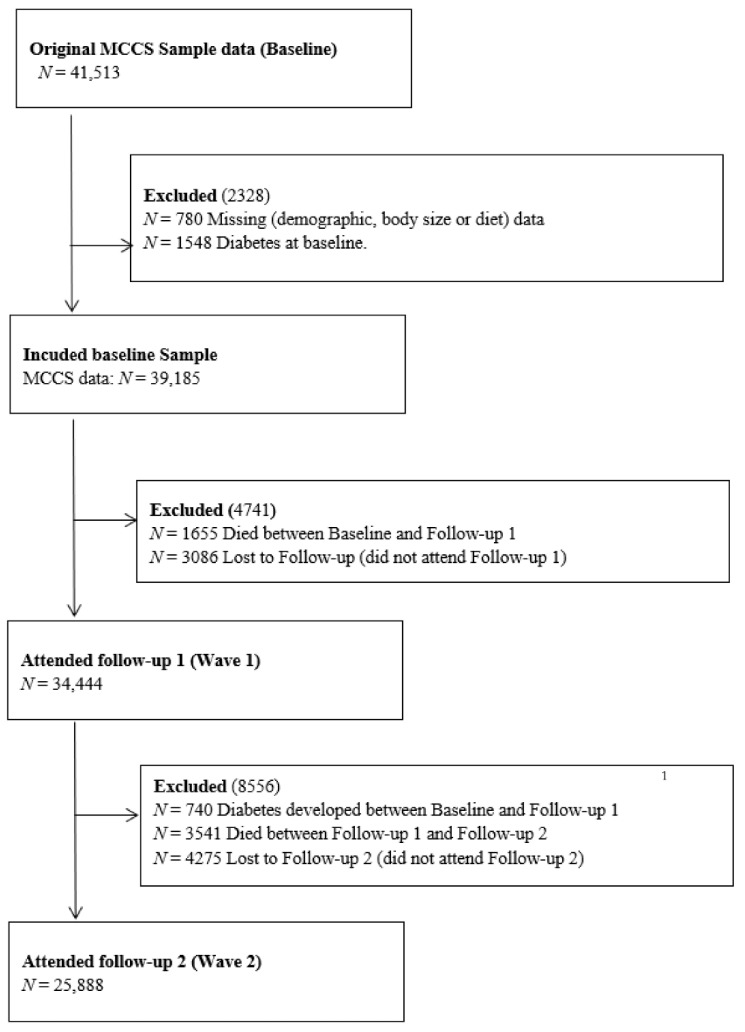
Participant flowchart. **^1^** People who had already developed diabetes between baseline and follow-up 1 were excluded after follow-up 1 to avoid double counting.

The MCCS is a prospective cohort with 41,513 participants living in Melbourne, recruited during the period between 1990 and 1994. Participants were recruited using the electoral roll and direct approach through clubs, churches and ethnicity-specific mass-media. The recruitment and follow-up of the cohort and data collection process has been detailed elsewhere [17]. Socio-demographic and dietary information was collected at baseline using interviewer-administered questionnaires. Anthropometric data were collected through physical measurements and blood samples collected. Follow-up surveys were conducted around 4 years after recruitment, between 1995 and 1998 (wave 1) and between 2003 and 2007 (wave 2). Of the 41,513 participants, 39,185 were eligible at baseline; of these, 34,444 were included in the first wave of follow-up and 25,888 were include in the second wave of follow-up (Figure 1). In the wave 1 follow-up data were collected either with a self-administered questionnaire or a questionnaire administered by interviewer over telephone. In the wave 2, the baseline information was updated using self-administered questionnaires and anthropometric (except height) measurements were repeated [17].

### 2.2. Dietary Assessment

A 121-item self-administered Food Frequency Questionnaire (FFQ) [18] was used to collect dietary intake data. Sex-specific average portion sizes were assigned to each food item and daily frequencies of some fruits were seasonally adjusted. Nutrient composition data were derived largely from the Australian food composition tables [19]. In the case of unavailability in Australian tables data were derived from relevant British (Folate and vitamin) [20], Royal Melbourne Institute of Technology data (Fatty acids) [21] and the United States Department of Agriculture (Carotenoids) [22] sources as appropriate. Mean daily nutrient intakes were obtained by multiplying the daily frequency of each food item by the nutrient composition for an average sex-specific portion size. Comparisons of antioxidant and fatty acid intakes against plasma biomarkers for this FFQ have been described previously [23,24]. Three diet scores were calculated from the estimated food and nutrient intakes. The DII was generated based on reviewing and scoring literature assessing the association between various dietary components including nutrients, spices and foods and six inflammatory biomarkers, giving a single score assessing anti- or pro-inflammatory potential of the overall diet [25]. DII values for MCCS cohort participants were calculated using 29 of 45 possible foods and nutrients. The higher the score the more pro-inflammatory the diet. The AHEI-2010 score is the sum of dietary component scores, ranging from 0 to 110 calculated for MCCS data using the method described by Chiuve et al., based on the scientific evidence available at the time on the association between diet and health [26]. The higher the score the healthier the diet. The Mediterranean Diet Score (MDS) assesses how closely the diet adheres to the traditional Cretan diet. The total score ranged from 0 to 9 with a higher score indicating stronger compliance with a traditional Mediterranean diet [27].

### 2.3. Socio Demographic and Comorbidity Data

Data on age, sex, country of origin, smoking, alcohol consumption and physical activity was collected using interviewer-administered questionnaires at baseline. According to country-of-origin participants were grouped into 1. Australia/New Zealand/other, 2. Northern European (primarily British) and 3. Southern European (Greek and Italian). Socioeconomic position was represented by deciles of Socio-Economic Indexes for Areas (SEIFA) Index of Relative Socio-economic Disadvantage based on postcode at baseline. Deciles of SEIFA were recoded into quintiles, the first being the most disadvantaged and the fifth being the most affluent. To assess physical activity, participants were asked how much time they spent on low, moderate and high levels of physical activity at home and at work, using a structured questionnaire. The responses were categorised as: none/week, 1–2 times/week; and ≥3 times/week, which were coded as 0, 1.5 and 4, respectively. An overall physical activity score was generated from the sum of scores from each category of activities, with high-intensity activity receiving double the weight of low-intensity activity and walking. The participant’s overall physical activity score was categorized into 4 approximate quartiles: 0; >0–4; >4–6 and >6. Self-reported health information covering diabetes and other comorbidities was collected.

### 2.4. Anthropometric Assessment Data

All anthropometric parameters (height, weight, waist and hip circumferences) were measured at baseline following standard procedures [17]. Weight was measured to 100 g using digital electronic scales, height to 1 mm using a stadiometer, and waist and hips circumferences were measured to 1 mm using a 2-m metal anthropometric tape. Weight and BMI and WHR calculated and classified as follows: BMI ≥ 25 kg/m^2^ (overweight), BMI ≥ 30 kg/m^2^ (obese), and WHR ≥ 0.90 cm (male); ≥0.85 cm (female) elevated risk.

### 2.5. Diabetes Ascertainment

At baseline people who reported having been diagnosed with diabetes or who had elevated blood glucose: fasting > 7.0 mmol/L or non-fasting > 11.0 mmol/L, were considered to have diabetes and were not eligible for the analysis. At both follow-up surveys participant were asked if they had been diagnosed with diabetes, if they answered yes to this question, they were considered to have incident diabetes.

### 2.6. Statistical Analysis

The DII and AHEI scores were divided into 5 groups of equal size using quintile cut points derived from the whole sample. The lowest of the groups representing the most anti-inflammatory/healthiest diet for DII and least healthy diet for AHEI. The MDS was classified into 3 categories with scores of 0–3; 4–6; and 7–9. Univariate associations of baseline characteristics and the dietary indices were evaluated using one-way ANOVA for continuous variables and chi-squared test for categorical variables. Cumulative incidences of diabetes at waves 1 and 2 were compared across predictor categories. Associations of baseline dietary indices [DII, AHEI and MDS) with incident diabetes adjusting for plausible confounders were assessed through fitting multivariable Poisson regression models [28] using robust error variance [29].

Incidence Rate Ratio (IRR) and 95% confidence intervals were generated fitting four graduated models. Model 1 was fitted adjusting for set of plausible confounding variables; Age, sex, SEIFA (quintiles 1–5), smoking status (never, former and current), drinking status (never, former and current), family history of diabetes and physical activity level (0, >0 and <4, ≥4 and <6, ≥6). Model 2 was fitted adjusting for variables of model 1 plus BMI, Model 3 was fitted adjusting for variables of model 2 plus WHR and model 4 additionally adjusted for region of origin. The interaction between dietary pattern and region of origin on the risk of type 2 diabetes was estimated by including a multiplicative term between the two variables in the generalized estimating equation models. As it was statistically significant, we examined the association of dietary pattern with type 2 diabetes stratified by region of origin.

In addition, first order interaction effects between dietary indices and sex, age, and WHR at baseline were investigated. Interaction effects between none of the pairs appeared significant (*p* > 0.05), and their inclusion did not improve model performance; therefore, only the main effects were retained in the final models. Trends across quantiles of dietary indices were estimated by giving each person in that quantile the median score for the quantile.

We fitted mediation models using body size (either BMI/WHR) as a mediator of the relationship between DII/AHEI and type 2 diabetes [30,31]. We did not include MDS in the mediation analysis, as there was no evidence of an association between MDS and diabetes. The controlled direct effect (CDE) is the average effect of an increase in DII/AHEI neither due to mediation nor interaction and the natural indirect effect (NIE) effect only due to mediation. The total effect is the product of the CDE and NIE [32,33]. The proportion mediated was calculated using the following formula [(CDE × (NIE − 1)]/[(CDE × NIE) − 1] [34].

All statistical analyses were performed using Stata/SE release 16 (Stata Corporation, College Station, TX, USA).

### 2.7. Ethics Approval

Cancer Council Victoria Human Research Ethics Committee approved the MCCS, and subjects gave their written informed consent to participate. The current study received approval from the Monash University Human Research Ethics Committee.

## 3. Results

Of the 41,513 participants recruited to the MCCS, 2328 (1548 with diabetes and 780 missing demographics, body size or diet data) were excluded. Of 39,185 included at baseline, 34,444 attended wave 1 (1655 died between baseline and wave 1 and 3086 were lost to follow-up). Among the wave 1 participants, 25,888 attended wave 2 (740 had already developed diabetes between wave 1 and wave 2, 3541 died between wave 1 and wave 2, 4275 did not attend wave 2) (Figure 1). The average age of the participants at baseline was 55.2 ± 8.7 years and 59.7% were female. Among the participants, 69.8% were born in Australia, 23.8% were of southern European origin and another 6.5% were of northern European origin.

At wave 1, participants with diets in the most pro-inflammatory quintile (Q5) of DII were more likely to be younger, female, disadvantaged (SEIFA Q1–Q3), of Southern European origin, smokers, less physically active (score < 4), have a higher WHR and BMI. Alcohol consumption showed an inverted U-shaped distribution (least in Q1 and Q5) across DII quintiles (Table 1a). Participants in the least adherent quintile (Q1) of AHEI-2010 were more likely to be male, disadvantaged (SEIFA Q1-Q3), smokers, never consume alcohol, less physically active (score < 4) and have higher WHR and BMI (Table 1b). Participants in the least adherent tertile (MDS 1) of MDS were more likely to be disadvantaged (SEIFA Q1–Q3), less physically active (score < 4) and have higher WHR and BMI. Family history of diabetes and proportion of people with comorbidities were evenly distributed across categories of dietary indices (Table 1c).

A total of 740 people had developed diabetes at wave 1 and an additional 1249 had developed diabetes by wave 2. The incidence of diabetes increased with increasing age, decreasing SEIFA and physical inactivity. The incidence of diabetes was higher among females, southern Europeans, smokers, those with higher BMI and WHR, positive family history, and one or more comorbidities. The lowest diabetes incidence was seen for current alcohol consumers. Diabetes incidence increased across DII quintiles and decreased across AHEI quintiles. The diabetes incidence varied little across MDS tertiles, revealing a U-shaped pattern. The trends were consistent across waves 1 and 2 of follow-up (Table 2).

Table 3 presents the associations of dietary indices at baseline with diabetes incidence from multi-variable Poisson regression models. Baseline DII was positively associated with diabetes incidence in model 1 after adjusting for age, sex, SEIFA, smoking status, drinking status, family history of diabetes and physical activity level (*p* trend < 0.001). Only quintile 5, the most pro-inflammatory diet, showed significant association after adjusting for BMI [IRR 1.25; 95% CI 1.08, 1.43, model 2; *p* trend = 0.005] and for BMI and WHR combined [IRR 1.21; 95% CI 1.05, 1.39, model 3, *p* trend 0.019]. However, after adjusting for region of origin (model 4) the association with DII became non-significant (*p* trend = 0.402). Only quintile 5 (most adherent diet quintile) of AHEI-2010 at baseline showed a significant association with diabetes [IRR 0.67; 95% CI 0.58, 0.78;] in model 1 and there was a significant trend (*p* < 0.001) across quintiles. The association persisted after adjustment for BMI [IRR 0.75; 95% CI 0.65, 0.87 *p* trend < 0.001] and additional adjustment for WHR [IRR 0. 78; 95% CI 0.67, 0.91; *p* trend = 0.003] and region of origin [IRR 0.73; 95% CI 0.63, 0.85; *p* trend < 0.001]. Baseline MDS score showed no significant association with diabetes risk in the multivariable Poisson regression models.

Table 4 presents the association of baseline dietary indices with diabetes, stratified by region of origin, adjusting for age, sex, SEIFA, smoking status, drinking status, family history of diabetes, and physical activity level, at baseline. Among the Australia and New Zealand born participants, baseline DII was positively associated with diabetes incidence [Q5 vs. Q1-IRR 1.49; 95% CI 1.22, 1.80; *p* trend < 0.001]. No significant association was seen in the participants of northern or southern European origin. Similarly, among the Australia and New Zealand born participants, baseline AHEI-2010 score was inversely associated with diabetes incidence after adjusting for confounders, [ Q5 vs. Q1- IRR 0.51; 95% CI 0.41, 0.62; *p* trend < 0.001]. No significant association was seen in the participants of northern or southern European origin. Baseline MDS score showed an inverse association with diabetes risk in the multivariable Poisson regression models for the Australian- and New Zealand-born group [Q3 vs. Q1- IRR 0.79; 95% CI 0.64, 0.98; *p* trend = 0.01]. After adjustment for BMI and WHR the associations were attenuated but still significant for DII and AHEI-2010 in the Australian and New Zealand group (data not shown).

**Figure 2:**
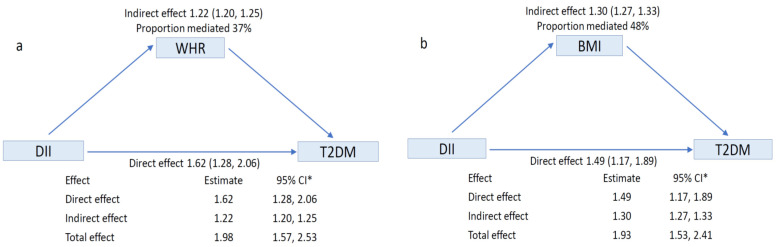

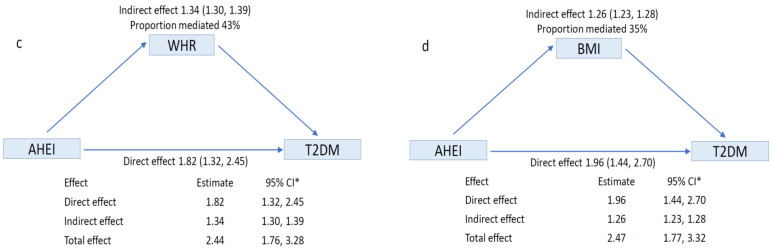
Mediation analyses of the association dietary pattern with risk of T2DM (**a**) DV—DII, IV—T2DM, MV—WHR. (**b**) DV—DII, IV—T2DM, MV—BMI. (**c**) DV—AHEI, IV—T2DM, MV—WHR (**d**) DV—AHEI, IV—T2DM, MV—BMI. Covariates for all mediation models—age, sex, socio-economic status, family history of T2DM, smoking status, alcohol intake and level of physical activity. Poisson regression model for direct effect model, linear regression for mediator model were fitted. The bias corrected 95% CI of the estimates were computed using the bootstrap resampling method with 1000 replications. * Statistically significant for *p* < 0.001.

Results of the mediation analyses are presented in Figure 2. Increasing DII was associated with increased diabetes risk, and 37% of the effect was explained by a substantial indirect effect through WHR, 48% of the effect was explained by a substantial indirect effect through BMI. Increasing AHEI was associated with lower diabetes risk, and 43% of the effect was explained by a substantial indirect effect through WHR, 35% of the effect was explained by a substantial indirect effect through BMI.

## 4. Discussion

In the MCCS, healthier diets defined by low inflammation potential or closer adherence to the US dietary guidelines were associated with lower risk of type 2 diabetes incidence. The MDS showed no clear association with diabetes risk. Associations for AHEI-2010 and DII were independent of BMI and WHR, but DII no longer showed an association after adjusting for region of origin. In analyses stratifying by country of origin, the associations for AHEI-2010 and DII were seen only in the Australian and New Zealand origin subgroup which made up 70% of the participants included in the analysis. Mediation analysis indicated that a substantial proportion of these associations was mediated through BMI or WHR.

We have previously investigated the prospective associations of the three diet scores DII, MDS, and AHEI-2010 and the development of obesity [16]. We found that healthier diets estimated by all three scores were associated with lower WHR at wave 2, while only DII and AHEI-2010 were associated with BMI. Overall, AHEI-2010 appeared to have the strongest association with body size measurements at wave 2. In the present study, AHEI-2010 and DII were associated with diabetes incidence independent of BMI and WHR, although the associations were attenuated after adjustment for BMI and less by the further addition of WHR. Our analyses showed that body size mediated between 35% and 48% of the association between diet scores and diabetes for both DII and AHEI-2010, somewhat lower than the 58% of association between an earlier version of DII and diabetes mediated by BMI in the E3N cohort [35]. There are several differences between the E3N and MCCS cohorts that could result in the various contributions of body size to the association between diet scores and diabetes risk, including that E3N participants were all women while the MCCS participants in our analysis were 40% men. However, we did not observe effect modification of the associations between diet scores and diabetes by sex in our study. Mean BMI in E3N was 23 kg/m^2^ while in the MCCS it was 27 kg/m^2^. Age at baseline was similar at 53 years for E3N and 55 years for MCCS.

The stratified analysis showed that for the Australia- and New Zealand-born participants the association between DII or AHEI-2010 and diabetes risk was strong, and consistent with the overall results, but there was no association in the European migrant subgroups. The northern European subgroup was relatively small, with around 2500 people and 100 cases, which may not have conferred sufficient statistical power to find associations. We have performed a validation study in the MCCS for the FFQ used at wave 2, finding that the performance of the FFQ was poorer for the southern European migrants than the Australian-born participants [36], which, if reflected in the baseline, FFQ reporting may contribute to the lack of association observed. It is also possible that because of the differences in diet between country-of-origin groups, the foods consumed for the same dietary scores are not consistent and may vary in their potential beneficial effects

Schwingshackl et al. [37] reviewed the association between 12 food groups that are the basis of many dietary indices: whole grains/cereals, refined grains/cereals, vegetables, fruits, nuts, legumes, eggs, dairy products, fish, red meat, processed meat, and sugar-sweetened beverages. Inverse associations were found for whole grain, dairy and fruit, and positive associations for processed meat, red meat and sugar sweetened beverages. Different weights for these different food groups in the scoring algorithms may explain the different associations between diet scores and diabetes. For example, Schwingshackl et al. found dairy to be inversely associated with diabetes risk, but in the MDS a point is allocated for having an intake of dairy products below the median, and sugar sweetened beverages are not scored in this index, which may contribute to the lack of association seen in our study for MDS.

Diabetes is understood to be a pro-inflammatory disorder [9], so healthy diets that reduce inflammation could potentially decrease risk. A Mediterranean diet has been shown to reduce circulating inflammatory biomarkers CRP, IL-6. TNF-α, and monocyte chemoattractant protein [12] and DII [38]. DII is, by design, correlated with inflammatory biomarkers [25] and this has been confirmed in numerous (i.e., over 35) different studies [39,40,41]. AHEI has also been shown to be inversely associated with inflammatory markers CRP and IL-6 [42], thus each of the diet indices assessed here may work to some degree through inflammatory pathways. In a previous study of MCCS participants we observed a moderate positive rank correlation between the MDS and the AHEI-2010 (Spearman ρ = 0.49), and moderate negative rank correlations for the DII with each of the MDS and the AHEI-2010 (ρ = −0.44, and ρ = −0.28, respectively) [42].

A major strength of our study is the use of a large dataset and a prospective study design with adjustment for many plausible confounders. We included 1989 incident cases of diabetes. Our study used three diet scores with different theoretical bases and reached similar conclusions for DII and AHEI-2010, with better diets associated with lower risk of diabetes. Also, we had anthropometric measurements rather than relying on self-reported measures often used in similar large datasets.

There were several limitations of our study. We used self-reported dietary data from an FFQ, which are known to measure intake with considerable error. Only 29 of 45 components of DII were available to be included for this study, which may limit comparison of the findings with other similar studies using different dietary variables. However, from over 350 peer reviewed publications, the average number of food parameters used to calculate the scores was 27. Physical activity data were self-reported, and the questions were not as detailed as in instruments commonly used today, such as IPAQ [43]. Diabetes diagnosis was also self-reported, although at the first follow-up, we confirmed the diagnosis with participant’s nominated doctor. Of doctors we could contact, 76% of incident diabetes cases were confirmed as type 2 diabetes [44]. We also assumed all incident cases were type 2 diabetes given the age of study participants. Diabetes diagnosis was not based on population screening therefore may be confounded by factors associated with health service usage, as data from the 2011–2012 Australian Health Survey suggests that there was approximately one newly diagnosed case of diabetes for every four diagnosed cases [45]. The numbers within country of birth strata were small and attendance at follow-up 2 was lower for Greek born participants who had the highest incidence of diabetes at the first follow-up [46].

## 5. Conclusions

Overall, our results were consistent with other prospective studies showing that better quality diets as assessed by various indices were associated with reduced diabetes risk. In the current study the association between dietary indices was mediated by body size but there was also a component of the association that was independent of the association with body size measures. As with our previous study looking at body size outcomes, the association appears to be slightly more consistent for AHEI-2010 than DII and there no association was seen for the MDS. AHEI may be a practical way to assess diet quality in relation to diabetes risk, further work to assess the use of AHEI-2010 to design dietary interventions in RCTs would be valuable.

## 6. Patents

Dr. Hebert reports grants and other from Connecting Health Innovations LLC (CHI), outside the submitted work; in addition, Dr. Hebert has IP protection via Federally registered trademark for the DII with royalties paid to the University of South Carolina by Connecting Health Innovations LLC (CHI) from DII-derived products. Dr. James R. Hébert owns controlling interest in Connecting Health Innovations LLC (CHI), a company that has licensed the right to his invention of the dietary inflammatory index (DII^®^) from the University of South Carolina in order to develop computer and smart phone applications for patient counselling and dietary intervention in clinical settings. Dr. Nitin Shivappa is an employee of CHI.

## Figures and Tables

**Table 1:** (**a**) Descriptive statistics by Dietary Inflammatory Index (DII) quintiles. (**b**) Descriptive statistics by Alternative Healthy Eating Index-2010 (AHEI) quintiles. (**c**) Descriptive statistics by Mediterranean Diet Score (MDS) quintiles.

(**a**)							
	**DII Q1**	**DII Q2**	**DII Q3**	**DII Q4**	**DII Q5**	**Total**	**Test Statistics**
DII median (IQR)	−2.9 (−3.3, −2.7)	−1.9 (−2.2, −1.7)	−1.0 (−1.2, −0.7)	−0.1 (−0.2, 0.4)	1.8 (1.3, 2.6)	−1.0 (−2.2, 0.4)	
Age							
<50 Years	2367 (30.1)	2512 (31.8)	2561 (32.4)	2645 (33.9)	2584 (33.4)	12,669 (32.3)	χ^2^ = 86.4; df = 8
50–59 Years	2436 (31.0)	2583 (32.7)	2576 (32.6)	2612 (33.5)	2586 (33.5)	12,793 (33.7)	*p* < 0.001
>=60 Years	3053 (38.9)	2803 (35.5)	2769 (35.0)	2539 (32.6)	2559 (32.6)	13,723 (35.0)	
Mean ± SD	55.9 ± 8.8	54.3 ± 8.7	55.1 ± 8.7	54.8 ± 8.6	54.8 ± 8.6	54.8 ± 8.5	*p* < 0.001
Gender							
Male	3461 (44.1)	3234 (41.0)	3113 (39.4)	3083 (39.6)	2900 (37.5)	15,791 (40.3)	χ^2^ = 76.2; df = 4
Female	4395 (55.9)	4664 (59.1)	4793 (60.6)	4713 (60.5)	4829 (62.5)	23,394 (59.7)	*p* < 0.001
SEIFA ^1^ Quintiles							
SEIFA Q1	1250 (15.9)	1236 (15.7)	1343 (17.0)	1499 (19.2)	1748 (22.6)	7076 (18.1)	χ^2^ = 529; df = 16 *p* < 0.001
SEIFA Q2	1382 (17.6)	1543 (19.5)	1604 (20.3)	1732 (22.2)	1888 (24.4)	8149 (20.8)	
SEIFA Q3	1207 (15.4)	1224 (15.5)	1213 (15.3)	1223 (15.7)	1304 (16.9)	6171 (15.8)	
SEIFA Q4	1584 (20.2)	1569 (19.9)	1473 (18.6)	1370 (17.6)	1258 (16.3)	7254 (18.5)	
SEIFA Q5	2433 (31.0)	2326 (29.5)	2273 (28.8)	1972 (25.3)	1531 (19.8)	10,535 (26.9)	
Region of Origin							
AUS/NZ ^2^/Other	6262 (79.7)	5936 (75.2)	5764 (72.9)	5160 (66.2)	4209 (54.5)	27,331 (69.8)	χ^2^ = 1900; df = 8 *p* < 0.001
Northern Europe	584 (7.4)	555 (7.0)	487 (6.2)	471 (6.0)	431 (5.6)	2528 (6.5)	
Southern Europe	1010 (12.9)	1407 (17.8)	1655 (20.9)	2165 (27.8)	3089 (40.0)	9326 (23.8)	
Smoking status							
Non-smoker	4690 (59.7)	4775 (60.5)	4598 (58.2)	4414 (56.6)	4237 (54.8)	22,714 (58.0)	χ^2^ = 67.2; df = 4
Smoker	3166 (40.3)	3123 (39.5)	3308 (41.8)	3382 (43.4)	3492 (45.2)	16,471 (42.0)	*p* <0.001
Alcohol dinking status							
Never	2289 (29.1)	2161 (27.4)	2136 (27.0)	2169 (27.8)	2386 (30.9)	11,141 (28.4)	χ^2^ = 40; df = 8
Former	808 (10.3)	819 (10.4)	846 (10.7)	813 (10.4)	813 (10.5)	4099 (10.5)	*p* < 0.001
Current	4759 (60.6)	4918 (62.3)	4924 (62.3)	4814 (61.8)	4530 (58.6)	23,945 (61.1)	
Physical activity score							
0	1185 (15.1)	1462 (18.5)	1637 (20.7)	1943 (24.9)	2343 (30.3)	8570 (21.9)	χ^2^ = 999; df = 12
>0 and <4	1399 (17.8)	1508 (19.01)	1634 (20.7)	1672 (21.5)	1672 (21.6)	7885 (20.1)	*p* < 0.0001
>=4 and <6	2939 (37.4)	2921 (37.0)	2829 (35.8)	2663 (34.2)	2577 (33.3)	13,929 (35.6)	
>=6	2333 (29.7)	2007 (25.4)	1806 (22.8)	1518 (19.5)	1137 (14.7)	8801 (22.5)	
WHR ^3^ at baseline							
Normal WHR	5290 (67.3)	5295 (76.0)	5248 (66.4)	4980 (69.3)	4696 (60.8)	25,509 (65.1)	χ^2^ = 105; df = 4
Raised WHR	2566 (32.7)	2603 (33.0)	2658 (33.6)	2816 (36.1)	3033 (39.2)	13,676 (34.9)	*p* < 0.001
BMI ^4^ at baseline							
<25	3305 (42.1)	3180 (40.3)	2910 (38.8)	2709 (34.8)	2357 (30.5)	14,461 (36.9)	χ^2^ = 432; df = 8
25–29.9	3296 (42.0)	3396 (40.30	3450 (43.6)	3401 (43.6)	3371 (43.6)	16,914 (43.2)	*p* < 0.001
>=30.0	1255 (16.0)	1322 (16.7)	1546 (19.6)	1686 (21.6)	2001 (25.9)	7810 (19.9)	
Mean ± SD	26.3 ± 4.1	26.5 ± 4.2	26.7 ± 4.3	27.1 ± 4.4	27.6 ± 4.7	26.8 ± 4.0	*p* < 0.001
Family history of diabetes							
No	6560 (83.5)	6507 (82.4)	6495 (82.2)	6415 (82.3)	6268 (81.1)	32,245 (82.3)	χ^2^ = 15.6; df = 4
Yes	1296 (16.5)	1391 (17.6)	1411 (17.9)	1381 (17.7)	1461 (18.9)	6940 (17.7)	*p* 0.004
Comorbidity							
No	3443 (43.8)	3563 (45.1)	3514 (44.5)	3622 (46.5)	3481 (45.0)	17,623 (45.0)	χ^2^ = 12.1; df = 4
Yes	4413 (56.2)	4335 (54.9)	4392 (55.6)	4174 (53.5)	4248 (55.0)	21,562 (55.0)	*p* 0.017
(**b**)							
	**AHEI Q1**	**AHEI Q2**	**AHEI Q3**	**AHEI Q4**	**AHEI Q5**	**Total**	**Test Statistics**
AHEI median (IQR)	50.5 (46, 53)	59 (57, 61)	65 (64, 66)	70.5 (69, 72)	78 (75.5, 82)	64.5 (57, 72)	
Age							
<50 Years	2789 (34.9)	2790 (32.8)	2329 (30.9)	2331 (31.3)	2430 (31.7)	12,669 (32.3)	χ^2^ = 41.9; df = 8
50–59 Years	2458 (30.8)	2746 (32.3)	2513 (33.4)	2505 (33.5)	2571 (33.5)	12,793 (32.6)	*p* < 0.001
>=60 Years	2747 (34.4)	2960 (34.8)	2692 (35.7)	2651 (35.4)	2673 (34.8)	13,723 (35.0)	
Mean ± SD	54.8 ± 8.8	55.1 ± 8.7	55.5 ± 8.6	55.4 ± 8.6	54.3 ± 8.5	55.2 ± 8.7	*p* < 0.001 *
Gender (Sex)							
Male	4592 (57.4)	3782 (44.5)	2751 (36.5)	2457 (32.8)	2209 (28.8)	15,791 (40.3)	χ^2^ = 1700; df = 4
Female	3402 (42.6)	4714 (55.5)	4783 (63.5)	5030 (67.2)	5465 (71.2)	23,394 (59.7)	*p* < 0.001
SEIFA ^1^ Quintiles							
SEIFA Q1	1739 (21.7)	1700 (20.0)	1334 (17.7)	1192 (15.9)	1111 (14.5)	7076 (18.0)	χ^2^ = 396; df = 16 *p* < 0.001
SEIFA Q2	1803 (22.6)	1791 (21.1)	1595 (21.2)	1529 (20.4)	1431 (18.7)	8149 (20.8)	
SEIFA Q3	1297 (16.2)	1367 (16.1)	1149 (15.2)	1228 (16.4)	1130 (14.7)	6171 (15.8)	
SEIFA Q4	1400 (17.5)	1543 (18.2)	1431 (19.0)	1389 (18.6)	1491 (19.4)	7254 (18.5)	
SEIFA Q5	1755 (22.0)	2095 (24.6)	2025 (26.9)	2149 (28.7)	2511 (32.7)	10,535 (26.9)	
Region of Origin							
AUS/NZ ^2^/Other	5835 (73.0)	5800 (68.3)	5008 (66.5)	5120 (68.4)	5568 (72.6)	27,331 (69.8)	χ^2^ = 191; df = 8
Northern Europe	446 (5.6)	524 (6.2)	477 (6.3)	489 (6.5)	592 (7.7)	2528 (6.4)	*p* < 0.001
Southern Europe	1713 (21.4)	2172 (25.6)	2049 (27.2)	1878 (25.1)	1514 (19.7)	9326 (23.8)	
Smoking status							
Non-smoker	3972 (49.7)	4838 (56.9)	4573 (60.7)	4691 (62.7)	4640 (60.5)	22,714 (58.0)	χ^2^ = 338; df = 4
Smoker	4022 (50.3)	3658 (43.1)	2961 (39.3)	2796 (37.3)	3034 (39.5)	16,471 (42.0)	*p* < 0.001
Alcohol dinking status							
Never	2332 (29.2)	2636 (31.0)	2532 (33.6)	2047 (27.3)	1594 (20.8)	11,141 (28.4)	χ^2^ = 394; df = 8
Former	944 (11.8)	870 (10.2)	757 (10.1)	736 (9.8)	792 (10.3)	4099 (10.5)	*p* < 0.001
Current	4718 (59.0)	4990 (58.7)	4245 (56.3)	4704 (62.8)	5288 (68.9)	23,945 (61.1)	
Physical activity score							
0	2251 (28.2)	2048 (24.1)	1687 (2.4)	1440 (19.2)	1144 (14.9)	8570 (21.9)	χ^2^ = 674; df = 12
>0 and <4	1650 (20.6)	1823 (21.5)	1500 (19.9)	1461 (19.5)	1451 (18.9)	7885 (20.1)	*p* < 0.0001
>=4 and <6	2637 (33.0)	2949 (34.7)	2759 (36.6)	2757 (36.8)	2827 (36.8)	13,929 (35.5)	
>=6	1456 (18.2)	1676 (19.7)	1588 (21.1)	1829 (24.4)	2252 (29.4)	8801 (22.5)	
WHR ^3^ at baseline							
Normal WHR	4149 (51.9)	5201 (61.2)	5026 (66.7)	5297 (70.8)	5836 (76.1)	25,509 (65.1)	χ^2^ = 1200; df = 4
Raised WHR	3845 (48.1)	3295 (38.8)	2508 (33.3)	2190 (29.3)	1838 (23.9)	13,676 (34.9)	*p* < 0.001
BMI ^4^ at baseline							
<25	2461 (30.8)	2883 (33.9)	2640 (35.0)	2955 (39.5)	3522 (45.9)	14,461 (36.9)	χ^2^ = 489; df = 8
25–29.9	3758 (47.0)	3769 (44.4)	3274 (43.5)	3132 (41.8)	2981 (38.9)	16,914 (43.2)	*p* < 0.001
>=30.0	1775 (22.2)	1844 (21.7)	1620 (21.5)	1400 (18.7)	1171 (15.3)	7810 (19.9)	
Mean ± SD	27.3 ± 4.4	27.1± 4.4	27.0 ± 4.4	26.6± 4.3	26.0 ± 4.5	26.8 ± 4.4	*p* < 0.001 *
Family history of diabetes							
No	6587 (82.4)	6980 (82.2)	6217 (82.5)	6168 (82.4)	6293 (82.0)	32,245 (82.3)	χ^2^ = 0.92; df = 4
Yes	1407 (17.6)	1516 (17.8)	1317 (17.5)	1319 (17.6)	1381 (18.0)	6940 (17.7)	*p* 0.922
Comorbidity							
No	3616 (45.2)	3782 (44.5)	3360 (44.6)	3311 (44.2)	3554 (46.3)	17,623 (45.0)	χ^2^ = 8.6; df = 4
Yes	4378 (54.8)	4714 (55.5)	4174 (55.4)	4176 (55.8)	4120 (53.7)	21,562 (55.0)	*p* 0.071
(**c**)							
	**MDS 1**	**MDS 2**	**MDS 3**	**Total**	**Test Statistics**		
MDS median (IQR)	3 (2, 3)	5 (4, 6)	7 (7, 8)	4 (3, 6)			
Age							
<50 Years	4411 (33.6)	6796 (32.0)	1462 (30.3)	12,669 (32.3)	χ^2^ = 20.1; df = 4		
50–59 Years	4187 (31.9)	7009 (33.0)	1597 (33.1)	12,793 (32.7)	*p* < 0.001		
>=60 Years	4538 (34.5)	7420 (35.0)	1765 (36.6)	13,723 (35.0)			
Mean ± SD	55.0 ± 8.7	55.2 ± 8.6	55.6 ± 8.6	55.2 ± 8.7	*p* < 0.001 *		
Gender							
Male	5389 (41.0)	8451 (39.8)	1951 (40.4)	15,791 (40.3)	χ^2^ = 4.97; df = 2		
Female	7747 (59.0)	12,774 (60.2)	2873 (59.6)	23,394 (59.7)	*p* 0.083		
SEIFA ^1^ Quintiles							
SEIFA Q1	2712 (20.7)	3690 (17.4)	674 (14.0)	7076 (18.10	χ^2^ = 272; df = 8		
SEIFA Q2	2931 (22.3)	4353 (20.5)	865(17.9)	8149 (20.8)	*p* < 0.001		
SEIFA Q3	2074 (15.8)	3384 (15.9)	713 (14.8)	6171 (15.8)			
SEIFA Q4	2323 (17.7)	3943 (15.6)	988 (20.50	7254 (18.5)			
SEIFA Q5	3096 (23.6)	5855 (27.6)	1584 (32.8)	10,535 (26.9)			
Region of Origin							
AUS/NZ ^2^/Other	9196 (70.0)	14,807 (69.8)	3328 (69.0)	27,331 (69.8)	χ^2^ = 43.8; df = 4		
Northern Europe	754 (5.7)	1368 (6.5)	406 (8.4)	2528 (6.5)	*p* < 0.001		
Southern Europe	3186 (24.3)	5050 (23.8)	1090 (22.6)	9326 (23.8)			
Smoking status							
Non-smoker	7552 (57.5)	12,392 (58.4)	2770 (57.4)	22,714 (58.0)	χ^2^ = 3.33; df = 2		
Smoker	5584 (42.5)	8833 (41.6)	2054 (42.6)	16,471 (42.0)	*p* 0.190		
Alcohol dinking status							
Never	4374 (33.3)	5945 (28.0)	822 (17.0)	11,141 (28.4)	χ^2^ = 496; df = 4		
Former	1396 (10.6)	2217 (10.5)	486 (10.1)	4099 (10.5)	*p* < 0.001		
Current	7366 (56.1)	13,063 (61.6)	3516 (72.9)	23,945 (61.1)			
Physical activity score							
0	3294 (25.1)	4486 (21.1)	790 (16.4)	8570 (21.9)	χ^2^ = 265; df = 6		
>0 and <4	2708 (20.6)	4244 (20.0)	933 (19.3)	7885 (20.1)	*p* < 0.001		
>=4 and <6	4621 (35.2)	7531 (35.5)	1777 (36.8)	13,929 (35.6)			
>=6	2513 (19.1)	4964 (23.4)	1324 (27.5)	8801 (22.5)			
WHR ^3^ at baseline							
Normal WHR	8275 (63.0)	13,967 (65.8)	3267 (67.7)	25,509 (65.1)	χ^2^ = 44.9; df = 2		
Raised WHR	4861 (37.0)	7258 (34.2)	1557 (32.3)	13,676 (34.9)	*p* <0.001		
BMI ^4^ at baseline							
<25	4617 (35.2)	7979 (37.6)	1865 (38.7)	14,461 (36.9)	χ^2^ = 57.3; df = 4		
25–29.9	5675 (43.2)	9115 (42.9)	2124 (44.0)	16,914 (43.2)	*p* < 0.001		
>=30.0	2844 (21.7)	4131 (19.5)	835 (17.3)	7810 (19.9)			
Mean ± SD	27.0 ± 4.5	26.8± 4.4	26.5 ± 4.1	26.8± 4.4	*p* < 0.001 *		
Family history of diabetes							
No	10,777 (82.0)	17,448 (82.2)	4020 (83.3)	32,245 (82.3)	χ^2^ = 4. 26; df = 2		
Yes	2359 (18.0)	3777 (17.8)	804 (16.7)	6940 (17.7)	*p* 0.119		
Comorbidity							
No	5894 (44.9)	9541 (45.0)	2188 (45.4)	17,623 (45.0)	χ^2^ = 0.348; df = 2		
Yes	7242 (55.1)	11,684 (55.0)	2636 (54.6)	21,562 (55.0)	*p* 0.084		

^1^ SEIFA Socio-Economic Indexes for Areas, ^2^ AUS/NZ Australia/New Zealand, ^3^ WHR waist to hip ratio, ^4^ BMI body mass index. * Statistically significant for *p* < 0.001.

**Table 2:** Cumulative incidence of diabetes in follow-up (wave) 1 and follow-up (wave) 2 across predictor categories.

Variables	Category	Wave 1		Wave 2	
		n (%)	*p* Value	n (%)	*p* Value
Age	<50 years	122 (1.1)	<0.001	276 (3.4)	<0.001
	50–59 years	263 (2.4)		499 (4.5)	
	>=60 years	355 (3.0)		474 (7.2)	
Sex	Male	376 (2.8)	<0.001	591 (6.9)	<0.001
	Female	356 (1.8)		658 (4.8)	
SEIFA ^1^	Q1	214 (3.6)	<0.001	282 (8.4)	<0.001
	Q2	188 (2.8)		289 (7.2)	
	Q3	111 (2.1)		198 (5.6)	
	Q4	98 (1.6)		221 (4.9)	
	Q5	129 (1.4)		268 (3.8)	
Region of Origin	AUS/NZ ^2^/Other	344 (1.5)	<0.001	734 (4.5)	<0.001
	Northern Europe	41 (1.9)		61 (4.0)	
	Southern Europe	355 (4.6)		454 (10.2)	
WHR ^3^ group	Normal	238 (1.1)	<0.001	503 (3.3)	<0.001
	High	502 (4.4)		746 (10.7)	
BMI ^4^ group	<25	63 (0.5)	<0.001	133 (1.5)	<0.001
	25–29.9	297 (2.1)		541 (5,7)	
	>=30	380 (5.8)		575 (14.5)	
Comorbidity	No	191 (1.2)	<0.001	403 (3.8)	<0.001
	Yes	549 (3.0)		846 (7.3)	
Family History	No	493 (1.8)	<0.001	865 (4.7)	<0.001
	Yes	247 (4.1)		384 (10.0)	
Smoking status	No	379 (1.9)	<0.001	699 (5.1)	<0.001
	Yes	361 (2.6)		550 (6.3)	
Drinking status	Never	276 (2.9)	<0.001	417 (7.0)	<0.001
	Former	91 (2.6)		150 (6.4)	
	Current	373 (1.8)		682 (4.8)	
Physical activity	0	266 (3.2)	<0.001	356 (7.7)	<0.001
	1–4	171 (2.5)		273 (5.9)	
	4–6	243 (2.0)		430 (5.6)	
	>=6	100 (1.3)		190 (3.5)	
DII ^5^	Q1	124 (1.8)	<0.001	199 (4.2)	<0.001
	Q2	133 (1.9)		246 (5.2)	
	Q3	144 (2.1)		251 (5.4)	
	Q4	154 (2.3)		262 (5.9)	
	Q5	185 (2.9)		291 (7.5)	
AEHI ^6^	Q1	175 (2.6)	<0.001	313 (7.4)	<0.001
	Q2	189 (2.6)		294 (6.2)	
	Q3	160 (2.5)		242 (5.6)	
	Q4	122 (1.9)		232 (5.3)	
	Q5	94 (1.4)		168 (3.6)	
MDS ^7^	0–3	268 (2.4)	0.118	444 (6.2)	0.003
	4–6	376 (2.1)		672 (5.5)	
	7–9	96 (2.3)		133 (5.8)	

^1^ SEIFA Socio-Economic Indexes for Areas, ^2^ AUS/NZ Australia/New Zealand, ^3^ WHR waist to hip ratio, ^4^ BMI body mass index, ^5^ DII Dietary Inflammatory Index, ^6^ AHEI Alternative Healthy Eating Index-2010, ^7^ MDS Mediterranean Dietary Score.

**Table 3:** Association of baseline dietary indices with diabetes adjusting for confounders.

	Adjusted ^1^ IRR (95% CI)	*p* Value	Adjusted ^2^ IRR (95% CI)	*p* Value	Adjusted ^3^ IRR (95% CI)	*p* Value	Adjusted ^4^ IRR (95% CI)	*p* Value
DII ^5^ Quintile								
DII Q1	Reference		Reference		Reference		Reference	
DII Q2	1.17 (1.01, 1.36)	0.03	1.14 (0.98, 1.31)	0.08	1.13 (0.98, 1.30)	0.11	1.10 (0.95, 1.27)	0.19
DII Q3	1.20 (1.05, 1.40)	0.01	1.11 (0.96, 1.27)	0.16	1.09 (0.95, 1.26)	0.23	1.06 (0.92, 1.22)	0.43
DII Q4	1.29 (1.11, 1.48)	0.001	1.14 (0.99, 1.31)	0.07	1.08 (0.96, 1.28)	0.15	1.05 (0.91, 1.21)	0.47
DII Q5	1.49 (1.30, 1.72)	<0.001	1.25 (1.08, 1.43)	0.002	1.21 (1.05, 1.39)	0.008	1.10 (0.95, 1.26)	0.21
*p* trend	<0.001		0.005		0.02		0.40	
AHEI ^6^ Quintile								
AHEI Q1	Reference		Reference		Reference		Reference	
AHEI Q2	0.97 (0.86, 1.10)	0.65	0.99 (0.88, 1.12)	0.87	1.02 (0.90, 1.15)	0.75	0.98 (0.87, 1.11)	0.77
AHEI Q3	0.94 (0.83, 1.07)	0.38	0.95 (0.84, 1.09)	0.59	0.99 (0.87, 1.13)	0.88	0.94 (0.83, 1.07)	0.34
AHEI Q4	0.87 (0.76, 1.00)	0.05	0.93 (0.81, 1.06)	0.28	0.96 (0.84, 1.10)	0.59	0.91 (0.80, 1.04)	0.18
AHEI Q5	0.67 (0.58, 0.78)	<0.001	0.75 (0.65, 0.87)	<0.001	0.78 (0.67, 0.91)	0.001	0.73 (0.63, 0.85)	<0.001
*p* trend	<0.001		<0.001		0.003		<0.001	
MDS ^7^ Category								
Score 0–3	Reference		Reference		Reference		Reference	
Score 4–6	0.93 (0.85, 1.02)	0.18	0.95 (0.86, 1.04)	0.24	0.95 (0.87, 1.04)	0.291	0.94 (0.86, 1.03)	0.20
Score 7–9	0.97 (0.84, 1.13)	0.69	0.99 (0.86, 1.15)	0.90	1.02 (0.88, 1.18)	0.836	0.98 (0.85, 1.13)	0.77
*p* trend	0.28		0.47		0.65		0.37	

^1^ Adjusted for age, sex, SEIFA, smoking status, drinking status, family history of diabetes and physical activity level at baseline, ^2^ Adjusted for all in 1 plus BMI, ^3^ Adjusted for all in 2 plus WHR, ^4^ Adjusted for all in 3 plus country of birth, ^5^ DII Dietary Inflammatory Index, ^6^ AHEI Alternative Healthy Eating Index-2010, ^7^ MDS Mediterranean Dietary Score.

**Table 4:** Association of baseline dietary indices with diabetes adjusting for confounders stratified by region of birth.

	Australia and New Zealand		Northern Europe		Southern Europe	
	Adjusted IRR (95% CI) ^1^	*p* Value	Adjusted IRR (95% CI) ^1^	*p* Value	Adjusted IRR (95% CI) ^1^	*p* Value
DII ^2^ Quintile						
DII Q1	Reference		Reference		Reference	
DII Q2	1.06 (0.92, 1.32)	0.27	1.63 (0.89, 2.99)	0.12	1.05 (0.80, 1.37)	0.74
DII Q3	1.11 (0.92, 1.33)	0.29	1.37 (0.71, 2.66)	0.35	1.12 (0.86, 1.45)	0.38
DII Q4	1.19 (0.99, 1.43)	<0.001	1.77 (0.96, 3.26)	0.07	1.02 (0.79, 1.31)	0.91
DII Q5	1.49 (1.22, 1.80)	<0.001	1.66 (0.87, 1.17)	0.13	1.02 (0.80, 1.30)	0.87
*p* trend	<0.001		0.13		0.75	
AHEI ^3^ Quintile						
AHEI Q1	Reference		Reference		Reference	
AHEI Q2	0.91 (0.77, 1.07)	0.25	1.39 (0.78, 2.47)	0.27	0.93 (0.76, 1.14)	0.50
AHEI Q3	0.75 (0.62, 0.89)	0.001	0.94 (0.49, 1.80)	0.84	1.10 (0.89, 1.36)	0.35
AHEI Q4	0.67 (0.55, 0.81)	0.05	0.87 (0.49, 1.71)	0.70	1.07 (0.87, 1.32)	0.55
AHEI Q5	0.51 (0.41, 0.62)	<0.001 *	0.90 (0.46, 1.79)	0.78	0.83 (0.65, 1.05)	0.13
*p* trend	<0.001		0.37		0.48	
MDS ^4^ Category						
Score 0–3	Reference		Reference		Reference	
Score 4–6	0.88 (0.77, 1.00)	0.047	1.05 (0.68, 1.62)	0.82	0.95 (0.82, 1.10)	0.47
Score 7–9	0.79 (0.64, 0.98)	0.033	0.91 (0.50, 1.69)	0.78	1.11 (0.90, 1.39)	0.33
*p* trend		0.011		0.94		0.80

^1^ Adjusted for Age, sex, SEIFA, smoking status, drinking status, family history of diabetes and physical activity level at baseline, ^2^ DII Dietary Inflammatory Index, ^3^ AHEI Alternative Healthy Eating Index-2010, ^4^ MDS Mediterranean Dietary Score. * Statistically significant for *p* < 0.001.

## Data Availability

Details on how to access data for the Melbourne Collaborative Cohort Study are available at: https://www.cancervic.org.au/research/epidemiology/pedigree.
